# Nitric Oxide and Electrophilic Cyclopentenone Prostaglandins in Redox signaling, Regulation of Cytoskeleton Dynamics and Intercellular Communication

**DOI:** 10.3389/fcell.2021.673973

**Published:** 2021-05-07

**Authors:** Ángel Bago, Miguel A. Íñiguez, Juan M. Serrador

**Affiliations:** ^1^Interactions with the Environment Program, Immune System Development and Function Unit, Centro de Biología Molecular “Severo Ochoa” (CBMSO), CSIC-UAM, Madrid, Spain; ^2^Departamento de Biología Molecular, Universidad Autónoma de Madrid, Madrid, Spain

**Keywords:** nitric oxide, cyclopentenone prostaglandins, S-nitrosylation, S-glutathionylation, actin cytoskeleton

## Abstract

Nitric oxide (NO) and electrophilic cyclopentenone prostaglandins (CyPG) are local mediators that modulate cellular response to oxidative stress in different pathophysiological processes. In particular, there is increasing evidence about their functional role during inflammation and immune responses. Although the mechanistic details about their relationship and functional interactions are still far from resolved, NO and CyPG share the ability to promote redox-based post-translational modification (PTM) of proteins that play key roles in cellular homeostasis, signal transduction and transcription. NO-induced S-nitrosylation and S-glutathionylation as well as cyclopentenone-mediated adduct formation, are a few of the main PTMs by which intra- and inter-cellular signaling are regulated. There is a growing body of evidence indicating that actin and actin-binding proteins are susceptible to covalent PTM by these agents. It is well known that the actin cytoskeleton is key for the establishment of interactions among leukocytes, endothelial and muscle cells, enabling cellular activation and migration. In this review we analyze the current knowledge about the actions exerted by NO and CyPG electrophilic lipids on the regulation of actin dynamics and cytoskeleton organization, and discuss some open questions regarding their functional relevance in the regulation of intercellular communication.

## Introduction

Nitric oxide (NO) and cyclopentenone prostaglandins (CyPGs) are inflammation-related agents of importance for cell and tissue homeostasis. NO is synthesized enzymatically from L-Arg by the action of nitric oxide synthases (neuronal: nNOS/NOS1, inducible: iNOS/NOS2 and endothelial: eNOS/NOS3). On the other hand, cyclooxygenase 1 (COX-1) and COX-2 catalyze the committed step in formation of prostaglandins (PGs) from arachidonic acid (AA) or dihomo-γ-linolenic acid (DGLA). Dehydration of PGs such as PGE_1_ and PGD_2_ results in the formation of CyPGs PGA_1_ and 15-deoxy-Δ12,14-PGJ_2_ (15-dPGJ_2_), respectively (Hobbs et al., 1999; Straus and Glass, 2001; Simmons et al., 2004; Oeste and Perez-Sala, 2014). Unlike precursor PGs, CyPGs are highly reactive bioactive lipids with anti-inflammatory, anti-tumoral, and anti-angiogenic features (Diez-Dacal and Perez-Sala, 2010; Surh et al., 2011). In the inflammatory process, NO and CyPGs can reach significant concentrations as a consequence of the inducible expression of NOS2 and COX-2, participating in modulation of the inflammatory process and immune response (Hernansanz-Agustin et al., 2013; Delmastro-Greenwood et al., 2014; Garcia-Ortiz and Serrador, 2018).

Evidence of a functional interplay between NO and PG has been reported; they often act simultaneously, with essential roles in similar pathophysiological conditions. Interaction between NO and PG occurs at multiple levels. NO can either stimulate or inhibit PG production depending on cell type, the source and levels of NO and on its selective activity on COX-1 or COX-2 expression (Salvemini et al., 2013). CyPG can also regulate the cellular levels of NO. In platelets and tumoral cells, selective inhibition of COX-2 reduces the synthesis of NO whereas in macrophages, 15-dPGJ_2_ reduces expression of iNOS either through inhibition of IKKβ or activation of PPAR-γ (Chen et al., 1997; Li et al., 2000).

Moreover, beyond the reciprocal regulation of NO and CyPG at the transcriptional level, these agents can also cross-talk by means of their ability to promote post-translational modification (PTM) of various proteins. These PTMs occur mainly through binding to redox sensitive Cys and can be mediated by NO-derived reactive nitrogen species (RNS) or by covalent thiol adduction of CyPG. There are many examples of proteins modified by both NO and CyPG: the transcription factor NF-κB; Ras; and Kelch-like ECH-associated protein 1 (Keap1), a sensor protein for oxidative stress that allows nuclear factor erythroid 2-related factor 2 (Nrf2) activation, promoting transcriptional activation and expression of antioxidant phase II genes (Straus et al., 2000; Cernuda-Morollon et al., 2001; Oliva et al., 2003; Renedo et al., 2007; Ibiza et al., 2008; Anta et al., 2016; Unoki et al., 2020).

Of note, another common target of NO and CyPG is the actin cytoskeleton. An increasing number of studies have identified actin and actin-binding proteins (ABPs) as major targets of RNS and CyPG in both physiological and oxidative pathophysiological conditions, resulting in alterations of actin network rearrangements by disturbing filament growth and stabilization (Gayarre et al., 2006; Horenberg et al., 2019; Varland et al., 2019).

In this review, we summarize the previous findings and more recent reports on the potential role of NO and CyPG in organization of the actin cytoskeleton and their involvement in regulation of cell signaling and gene expression during intercellular communication. We discuss the mechanisms by which these agents exert their functions, focusing on the interaction among leukocytes, endothelial and muscular cells as the paradigm of how NO and electrophilic CyPG may regulate homocellular and heterocellular communication in the immune and vascular systems.

## No- and CyPG-Mediated Post-Translational Modifications of Actin

**FIGURE 1:**
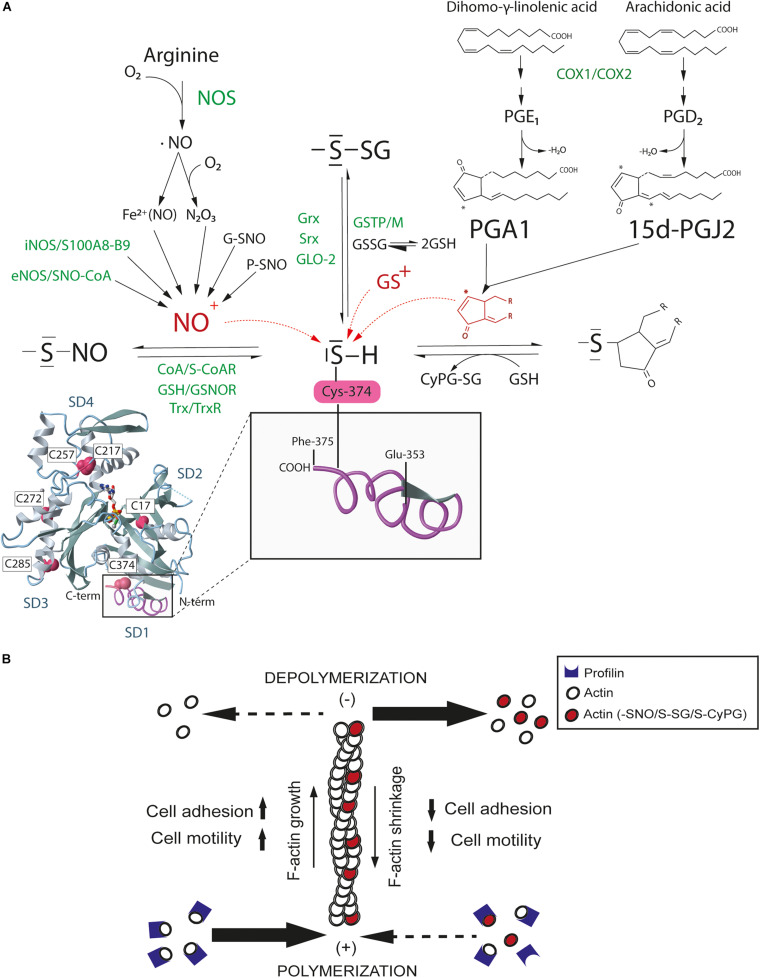
S-nitrosylation, S-glutathionylation, and CyPG adduction of actin Cys374. **(A)** Nitric oxide (NO) produced by NO synthases (NOS) spontaneously and enzymatically S-nitrosylates actin. S-nitrosylation mainly occurs through the electrophilic attack of the nitrosonium cation (NO^+^) on the sulfhydryl group of Cys, a reaction reversed by denitrosilases. Oxidized glutathione (GSSG) and glutathione-S-transferases (GSTP-M) glutathionylate actin whereas some reductases (e.g., Grx) promote actin deglutathionylation. Actin forms adducts with PGA_1_ and 15-dPGJ_2_, which can be reduced by glutathione (GSH). Spontaneous (black) and enzymatic (green) processes are shown. In red, electrophilic NO+, glutathionyl (GS+) and cyclopentenyl ring reactive species against the nucleophilic Cys sulfhydryl group of actin for S-nitrosylation, S-glutathionylation and CyPG adduction. Monomeric actin structure with subdomains (SD1-4) and redox sensitive Cys has been modified from MMDB ID:67242 using the NCBI Molecular Modeling Database (Madej et al., 2014). The inset shows the C-terminal region of actin in subdomain 1 (SD1) from Glu353 to Phe375. **(B)** Graphical abstract of actin filament shortening by redox-based post-translational modifications. S-nitrosylation, S-glutathionylation, and CyPG adduction of actin may disturb cell adhesion and motility likewise by reducing actin binding to profilin and polymerization at the barbed-end (+) and/or enhancing actin depolymerization from the pointed-end (–), which eventually induces F-actin shrinkage and actin cytoskeleton reorganization.

The best-recognized pathway for NO mediated cell signaling is through binding to the heme prosthetic group of soluble guanylate cyclase and the activation of protein kinase G by cGMP. However, NO also mediates other biologically significant actions by redox-based PTM including S-nitrosylation and S-glutathionylation of Cys (Figure 1A), interrelated reversible processes that can occur both spontaneously and enzymatically (Martinez-Ruiz et al., 2011; Seth et al., 2018; Wolhuter et al., 2018). S-nitrosylation can take place enzymatically through a complex formed between iNOS and the S100 calcium binding proteins A8 (S100A8) and A9 (S100A9), and also through S-nitroso-CoA, which can transfer the nitrosative activity of NO from eNOS (Jia et al., 2014; Zhou et al., 2019). S-nitrosylated proteins become denitrosylated by thioredoxin (Trx/TrxR), S-nitrosoglutathione (GSH/GSNOR) and S-nitroso-CoA (SNO-CoA-SCOR) reductase systems (Garcia-Ortiz and Serrador, 2018). S-glutathionylation occurs by mixed disulfide bonds between glutathione (GSH) and Cys. Glutathione transferase (e.g., GSTP1-1 and GSTM1-1) and glutaredoxin (Grx1 and Grx2) are the main systems of enzymatic glutathionylation and deglutathionylation, respectively. S-nitrosylated proteins can also react with GSH and become denitrosylated by a nitrosyl to glutathionyl radical shift. This reaction competes *in vivo* with transnitrosylation, the transference of nitrosyl groups between proteins, producing denitrosylated proteins and free GSNO, which, in turn, can either glutathionylate or S-nitrosylate other proteins (West et al., 2006). Likewise, electrophilic CyPG can signal in cells by covalent binding to the nucleophilic thiol group of redox-sensitive Cys, a reaction that takes place through Michael addition of the α,β-unsaturated carbonyl group in the cyclopentane ring of the CyPG (Oeste and Perez-Sala, 2014; Figure 1A).

Proteomics studies have consistently shown that actin can be modified by S-nitrosylation, S-glutathionylation and adduct formation with the CyPGs PGA_1_ and 15-dPGJ_2_ in a remarkable number of tissues and cell types expressing β and γ non-muscular actin (e.g., leukocytes and endothelial cells) or α muscular actin (e.g., smooth muscle cells) (Fratelli et al., 2002; Gayarre et al., 2006; Nardo et al., 2009; Su et al., 2013).

S-nitrosylation of actin promotes changes in actin polymerization, and Cys217, 257, 272, 285, and 374 have all been identified as targets of S-nitrosylation in muscle cells and neutrophils. These Cys residues are located nearest to the C-terminus of actin, an important region for the establishment of actin inter-monomer contacts and association with profilin, gelsolin and cofilin among other regulatory ABPs (Thom et al., 2008; Su et al., 2013). Moreover, a number of ABPs are also S-nitrosylated on redox-sensitive thiols. Thus, the interaction of actin with cofilin, α-actinin, drebrin, filamin, tropomyosin, and plastin/fimbrin may be regulated by S-nitrosylation (Martinez-Ruiz and Lamas, 2005; Ulrich et al., 2012; Figueiredo-Freitas et al., 2015). Ezrin and moesin, which are plasma membrane organizers that link the actin cytoskeleton to the cytoplasmic tail of transmembrane proteins, are also S-nitrosylated on Cys117 and 284 in response to iNOS-derived NO, increasing actin cytoskeleton tension and cell migration (Fernando et al., 2019; Zhang et al., 2019).

Actin is also a target for S-glutathionylation on Cys17, 217, and 374 (Wang et al., 2001; Hamnell-Pamment et al., 2005). S-glutathionylation impairs actin polymerization and destabilizes F-actin, in part by reducing the interaction between actin and tropomyosin, which triggers morphological changes that are reverted by Grx-mediated deglutathionylation (Pastore et al., 2003; Wang et al., 2003). Glyoxilase II (Glo2), sulfiredoxin 1 (Srx1) and protein disulfide isomerase (PDI) are other redox enzymes that could catalyze deglutathionylation of actin in eukaryotic cells (Findlay et al., 2006; Sobierajska et al., 2014; Ercolani et al., 2016). Interestingly, S-glutathionylation of the elastic protein titin on Cys47 and 63 at the F-actin-rich sarcomere regulates stiffness in both skeletal and cardiac muscle through inhibition of titin refolding (Herrero-Galan et al., 2019).

CyPGs are also redox regulators of actin. Several studies have shown that, PGA_1_ and 15-dPGJ_2_ bind covalently to actin on Cys374, inducing morphological changes in the actin cytoskeleton of neuroblastoma and mesangial cells by interfering with the formation of F-actin, with less abundant filaments, shorter length and altered structure (Gayarre et al., 2006; Stamatakis et al., 2006; Aldini et al., 2007). CyPG also regulate the interaction of actin with vimentin. PGA_1_ and 15-dPGJ_2_ directly bind to vimentin on Cys328, promoting cytoskeletal rearrangements that impair mitosis progression by interfering with its association with actin at the cell cortex (Duarte et al., 2019; Monico et al., 2019). In this regard, although both S-nitrosylation and S–glutathionylation on Cys328 have been reported to affect vimentin elongation (Kaus-Drobek et al., 2020), whether some of these redox-based PTMs disrupt the association between vimentin and actin at the cell cortex remains unknown.

Therefore, a large body of evidence supports Cys374 as the residue most sensitive to redox in actin, undergoing mild oxidation by RNS, GSH/GSSG or CyPG. The high reactivity of Cys374 may be in part due to its position as the penultimate amino acid residue in the C-terminal domain of actin, leaving it to be partially solvent-exposed and thus accessible to PTM. In addition, its proximity to Tyr133 (aromatic ring) and Arg116 (basic) in actin subdomain 1 would favor the reactivity of the Cys374 sulfhydryl group (Lassing et al., 2007). Moreover, Cys374 contributes to F-actin intra- and inter-strand contacts between actin subunits, fostering actin polymerization and F-actin stability (Oda et al., 2009; Dominguez and Holmes, 2011). In this regard, S-nitrosylation, S-glutathionylation and CyPG adduction on Cys374 similarly disturb the organization of the actin cytoskeleton, reducing cell adhesion and motility (Figure 1B). Nevertheless, the functional relationship between these actin PTMs remains an open question. It is plausible that S-nitrosylation and CyPG adduction of Cys374 do not represent end-effector PTMs but rather intermediate stages of actin oxidation, as observed previously (Giustarini et al., 2005; Gayarre et al., 2007; Wolhuter and Eaton, 2017). Changes in the GSH/GSSG ratio may switch these PTMs to glutathionylation, as a protective modification to preserve actin functions from harmful nitrosative and oxidative stress.

## No- and CYPG-Mediated Redox Post-Translational Modifications in Endothelial Cell Junctions

**FIGURE 2:**
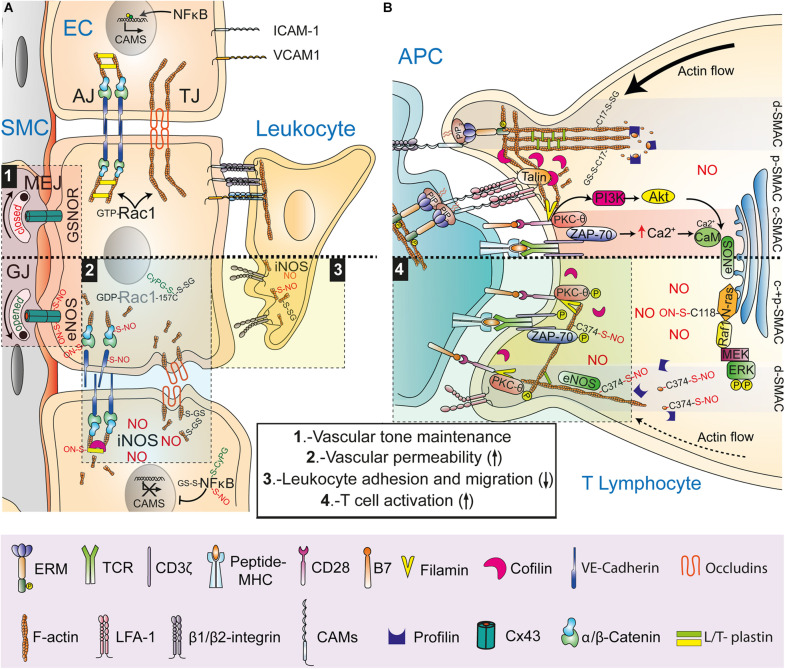
NO- and CyPG-mediated redox post-translational modifications of actin at cell-cell interactions. **(A)** Regulation of endothelial cell junctions and leukocyte-endothelial cell interactions. Upper part, Rac-1-mediated formation of actin-based adherens junctions (AJ) and tight junctions (TJ), NF-κB-promoted induction of cell adhesion molecules (CAMs) expression on activated endothelial cells (EC), integrin-mediated formation of leukocyte-EC docking structures, and closure of gap junctions (GJ) by GSNOR-mediated Cx45 denitrosylation at myoendothelial junctions (MEJ) are depicted. Lower part, Nitric oxide (NO) synthesized by eNOS opens GJ to control vascular SMC tone through S-nitrosylation of Cx45 (1) and disrupts AJ through S-nitrosylation of β-catenin and VE-cadherin increasing vascular permeability (2). Rac-1 inhibition by S-glutathionylation and CyPG adduction on Cys157 interferes with AJ and TJ formation whereas iNOS-derived NO interferes with AJ and TJ formation through cofilin activation by S-nitrosylation of T-plastin and S-glutathionylation of actin, respectively (2). NO produced by iNOS in ECs downregulates CAMs by either S-nitrosylation, S-glutathionylation, or CyPG adduction of NF-κB and in leukocytes interferes with β2 integrin activation by actin S-nitrosylation and S-glutathionylation, reducing leukocyte adhesion to ECs and migration (3). **(B)** Regulation of the immune synapse (IS). Upper part, retrograde actin flow in a mature IS showing ICAM-associated ERM, L-plastin-bundled F-actin and actin S-glutathionylated on Cys17 at the d-SMAC; cofilin and clusters of talin-associated LFA-1 at the p-SMAC; and signaling complexes between filamin-associated CD28 and PKC-θ and between TCR and ZAP-70 at the c-SMAC. Lower part, reduced retrograde actin flow upon full eNOS activation on the Golgi complex and the d-SMAC, where actin binding to profilin and polymerization is disturbed by S-nitrosylation on Cys374. Inactivated LFA-1 and mislocalization of cofilin are depicted. Enhanced phosphorylation of dispersed CD28-filamin-PKC- and TCR-ZAP-70 microclusters increases T cell activation (4).

Regulation of F-actin dynamics is essential for the endothelial barrier function of the adherens junctions (AJs). Homophilic extracellular adhesion between vascular endothelial (VE)-cadherin molecules and anchorage to the actin cytoskeleton through α-/β-catenin complexes associated with the cytoplasmic tail of VE-cadherin are needed to stabilize AJs between endothelial cells. Recent studies have shown that S-nitrosylation regulates AJ and vascular permeability at different levels (Figure 2A). Activation of endothelial cells with platelet-activating factor (PAF) S-nitrosylates VE-cadherin solely on its transmembrane-localized Cys, promoting VE-cadherin phosphorylation on Tyr, dissociation from actin-cytoskeleton–linked β-catenin and internalization (Guequen et al., 2016). Moreover, β-catenin is S-nitrosylated on Cys619 in response to eNOS activation by VEGF, PAF, or TNF-α, inducing dissociation from VE-cadherin (Thibeault et al., 2010; Marin et al., 2012). Release of F-actin-linked α-/β-catenin complexes from VE-cadherin increases permeability through the dissociation of the actin cytoskeleton from the AJ. S-nitrosylation of T-plastin also disturbs AJs; angiotensin-II-induced iNOS expression in aortic endothelial cells S-nitrosylates T-plastin on Cys566, increasing the F-actin-severing activity of cofilin at the AJ, which may be involved in the pathophysiological basis of thoracic aortic dissection, a disease in which aortic tissues from patients show high levels of S-nitrosylation (Pan et al., 2020). Rac-1, a small Rho GTPase of importance for the actin cytoskeletal rearrangements that orchestrate AJ formation in endothelial cells, is also a redox target of S-nitrosylation, S-glutathionylation and 15-dPGJ_2_ adduction. S-glutathionylation and 15-dPGJ_2_ adduction on Cys157 inhibits actin polymerization by Rac-1, disrupting the AJ and increasing endothelial permeability, whereas S-nitrosylation on Cys157 has also been observed in muscular cells, although the functional relevance of this PTM remains unknown (Su et al., 2013; Wall et al., 2015; Han et al., 2016). In contrast, *in vitro* studies with GSSG have shown that Rac-1 can be S-glutathionylated on Cys18, a PTM that accelerates nucleotide exchange and activation (Hobbs et al., 2015).

Tight junctions (TJ) and gap junctions (GJ) are also targets of oxidative and nitrosative stress. In Friedich ataxia, frataxin deficiency increases S-glutathionylation of actin, disrupting actin cytoskeleton anchorage to TJ proteins in micro VE cells of the blood–brain barrier, a PTM that is also characteristic of aortic valve sclerosis in humans (Valerio et al., 2019; Smith and Kosman, 2020). Coordinated S-nitrosylation/denitrosylation of GJ connexin (Cx)43 on Cys271 by compartmentalized activation of eNOS and GSNOR on the endothelial side of myoendothelial junctions (MEJ), controls smooth vascular muscle contraction and relaxation (Straub et al., 2011). The pathophysiological significance of Cx43 S-nitrosylation is illustrated in a mouse experimental model of Duchenne muscular dystrophy in which the opening of Cx43 hemichannels by NO-mediated S-nitrosylation led to cardiac stress-induced arrhythmias (Lillo et al., 2019).

Therefore, ample evidence exists to consider endothelial cell junctions as hot-spots of oxidative and nitrosative stress, with actin cytoskeleton-associated proteins anchored to them as important redox targets for the regulation of leukocyte homeostasis and inflammation.

## No- and CyPG-Mediated Redox Post-Translational Modifications in Leukocyte-Endothelial Interactions

In the vascular system, NO and CyPG regulate leukocyte adhesion to endothelial cells, thus working as anti-inflammatory agents. Expression of the adhesion molecules ICAM-1, VCAM-1, and E- and P- selectin on activated endothelial cells is modulated at the transcriptional level by RNS- and CyPG-mediated PTM of NF-κB. S-nitrosylation, glutathionylation and 15-dPGJ_2_ adduction of IKKβ, p50, and p65 reduce the transcriptional activity of NF-κB by targeting Cys62, 38, and 179, respectively (Straus et al., 2000; Cernuda-Morollon et al., 2001; Pineda-Molina et al., 2001; Castrillo et al., 2003; Reynaert et al., 2004, 2006; Lin et al., 2012). However, the regulation exerted on leukocyte-endothelial interactions by NO and CyPG is not restricted to endothelial cells (Figure 2A). For instance, 15-dPGJ_2_ abrogates ICAM-1 expression on endothelial cells in mesenteric vessels through an NO-dependent mechanism, but disturbs actin cytoskeleton rearrangements and migration in renal carcinoma cells and neutrophils likely through formation of adducts with actin (Aldini et al., 2007; Napimoga et al., 2008; Yamamoto et al., 2020). Similarly, S-nitrosylation, and S-glutathionylation of actin regulate adhesion to endothelial cells and migration of neutrophils by interfering with cytoskeleton dynamics. In this way, hyperoxia- and hyperglycemia-induced nitrosative stress by iNOS-synthesized NO S-nitrosylates β-actin on Cys 257, 272, 285, and 374, leading to a subset of short actin filaments that bind NLRP3 and disturbs β_2_ integrin clustering and adhesion of neutrophils to ICAM-1, which activates inflammasomes (Thom et al., 2008, 2017). This process is transient and followed by association of FAK, Trx/TrxR, VASP, and Rac with S-nitrosylated actin for subsequent denitrosylation and reorganization of F-actin to restore clustering and activation of β_2_ integrins (Thom et al., 2012, 2013). Impairment of adhesion via β_2_ integrins has also been reported in N-formylmethionyl-leucyl-phenylalanine (fMLF)-mediated migration of neutrophils from Grx1-deficient mice, which show reduced actin polymerization and increased S-glutathionylation of actin on Cys374, a PTM that also occurs upon oxidative stress by NADPH oxidase (Sakai et al., 2012).

Therefore, dynamic coordination between actin S-nitrosylation and S-glutathionylation seems necessary for the actin cytoskeleton rearrangements that regulate neutrophil chemotaxis.

## S-Nitrosylation and S-Glutathionylation at the T Cell Immune Synapse

S-nitrosylation also plays a key role in the regulation of T cell cognate interactions. The development of T lymphocytes is compromised in GSNOR-deficient mice whereas induction of GSNOR expression increases T cell activation by reducing S-nitrosylation of Akt on Cys224, which allows phosphorylation on Ser473 and Akt signaling (Yang et al., 2010; Li et al., 2018). On the other hand, compartmentalized production of NO by eNOS on the Golgi complex of T cells stimulated with antigen-pulsed antigen-presenting cells (APCs) activates N-Ras by S-nitrosylation on Cys118, leading to Ras-Raf-MEK-ERK signaling and activation-induced cell death (AICD) (Ibiza et al., 2008). Despite the ability of S-nitrosylation to regulate signaling targets downstream of the T cell receptor (TCR), a more general mechanism involving S-nitrosylation of actin for regulation of TCR-triggered signal transmission at the immune synapse (IS) can be considered (Figure 2B). NO synthesized by eNOS near the F-actin ring at the distal supramolecular activation clusters (d-SMAC) disturbs the coalescence of signaling microclusters of CD3, PKC-θ and CD28 in the central (c-)SMAC. These molecules show increased phosphorylation, which leads to a stronger activation of NF-κB (Ibiza et al., 2006; Garcia-Ortiz et al., 2017). Changes exerted by NO on signaling microclusters depend on the S-nitrosylation of actin on Cys374, which interferes with the formation of profilin-actin complexes, the main cellular source of ATP-activated actin used by formins and Arp 2/3 to nucleate filaments (Garcia-Ortiz et al., 2017). By interfering with binding between profilin and actin, S-nitrosylation reduces actin polymerization and retrograde flow toward the c-SMAC, the proposed main force driving movement of microclusters of TCR-associated signaling receptors, kinases and adaptors from the d-SMAC to the c-SMAC, where they dissociate from the TCR and signaling is attenuated (Hammer et al., 2019).

S-glutathionylation also plays an important function in T cell activation and IS formation. Sulphoraphane and piperlongumine, natural plant-derived prooxidative compounds that lower intracellular GSH levels, reduce the S-glutathionylation of actin, the expression of the activation markers CD69 and CD25, the secretion of IL-2 and T cell proliferation. Low GSH inhibits differentiation of proinflammatory Th17 but not Th1 and Th2 cells by reducing the production of IL-22, IL-17A, and IL-17F through inhibition of the transcription factors RORγt, HIF-1α, and STAT-3 (Liang et al., 2018, 2020). Interestingly, depletion of GSH by piperlongumine interferes with the enrichment of CD3 and the β_2_ integrin LFA-1 at the c- and peripheral (p)-SMAC, respectively, switching S-glutathionylation of actin on Cys17 to irreversible sulfinylation and sulfonylation (Liang et al., 2020).

In addition, some evidence suggests that S-nitrosylation and/or glutathionylation may also regulate the function of ABPs in the T cell IS. Cofilin, an F-actin severing ABP localized at the p-SMAC, is a target of oxidative stress and RNS. H_2_O_2_ impairs cofilin function through formation of an intramolecular disulfide bridge between Cys39 and 80. This PTM inhibits dephosphorylation-induced activation of cofilin on Ser3 and disturbs F-actin depolymerization in the IS, which is necessary for localization of LFA-1 and cofilin itself at the p-SMAC. On the other hand, S-nitrosylation and S-glutathionylation of cofilin on both Cys80 and 139 by eNOS-derived NO and by expression of GSTP1, respectively, have been described to reduce the F-actin-severing activity of cofilin (Zhang et al., 2015; Kruyer et al., 2019). L-plastin, an F-actin-bundling ABP whose phosphorylation on Ser5 facilitates the actin-dependent organization of LFA-1 at the p-SMAC (Wabnitz et al., 2010; Wang et al., 2010), also forms an intramolecular disulfide bridge between Cys42 and 101 when T cells are exposed to oxidative stress with H_2_O_2_. Oxidation of L-plastin is reversed by the Trx/TrxR system, which targets Cys101 as the most redox sensitive Cys residue of L-plastin in T cells (Balta et al., 2019). In this regard, recent reports have shown that iNOS-mediated nitrosative oxidation in neutrophils induces S-glutathionylation of L-plastin on Cys460, leading to the compartmentalized dissociation of F-actin bundles at the leading edge (Dubey et al., 2015). The actions that these NO-mediated cofilin and plastin PTMs exert on the reorganization of the actin cytoskeleton raises the possibility that other ABPs localized within the IS and susceptible to S-nitrosylation/S-glutathionylation (e.g., α-actinin, drebrin, dynamin, catenin or ezrin) may also work as redox sensors for the regulation of T cell activation.

## Conclusion

Increasing evidence demonstrates that RNS- and CyPG-mediated redox PTMs on reactive Cys residues promote changes in the structure and function of actin cytoskeleton regulatory proteins thus playing an essential role in cell function. In physiological conditions, NO is produced at basal steady-state levels, and reversible S-nitrosylation/denitrosylation controls cell homeostasis. However, higher levels of NO and PGs are released simultaneously in inflammatory conditions, mainly due to the inducible expression of iNOS and COX-2, respectively. RNS- and CyPG-mediated PTM contribute to the actin cytoskeleton-associated outcome of intercellular interactions between immune and vascular cells, controlling leukocyte activation, migration and vascular permeability (Figure 2). This is of importance from a pathophysiological perspective, as NO and CyPGs are redox regulators of development and resolution of the inflammatory process. How reversibility of S-nitrosylation and CyPG adduction is preserved during increasing inflammation-induced oxidative stress is still an unanswered question. In this regard, glutathionylation is now also considered to be a physiologically important PTM by which intracellular redox changes are transduced into functional responses (Klatt and Lamas, 2000). Although the mechanisms involved in protein glutathionylation are not yet fully understood, they may take place in part through RNS- and CyPG-mediated PTMs as intermediate products of oxidation on protein thiols. A better understanding of how NO- and CyPG–mediated PTMs cooperate to regulate actin polymerization and stability may provide new insights into the regulation of vascular dysfunction and inflammatory immune responses.

## Author Contributions

All authors listed have made a substantial, direct and intellectual contribution to the work, and approved it for publication.

## Conflict of Interest

The authors declare that the research was conducted in the absence of any commercial or financial relationships that could be construed as a potential conflict of interest.
